# (Immuno)proteasomes as therapeutic target in acute leukemia

**DOI:** 10.1007/s10555-017-9699-4

**Published:** 2017-10-25

**Authors:** Jacqueline Cloos, Margot SF Roeten, Niels E Franke, Johan van Meerloo, Sonja Zweegman, Gertjan JL Kaspers, Gerrit Jansen

**Affiliations:** 1 Departments of Pediatric Oncology/Hematology, VU University Medical Center, Amsterdam, The Netherlands; 2 Departments of Hematology, VU University Medical Center, Amsterdam, The Netherlands; 3 Princess Màxima Center, Utrecht, The Netherlands; 4 Amsterdam Rheumatology and Immunology Center, VU University Medical Center, Amsterdam, The Netherlands

**Keywords:** Leukemia, Constitutive proteasome, Immunoproteasome, Proteasome inhibition, Drug resistance

## Abstract

The clinical efficacy of proteasome inhibitors in the treatment of multiple myeloma has encouraged application of proteasome inhibitor containing therapeutic interventions in (pediatric) acute leukemia. Here, we summarize the positioning of bortezomib, as first-generation proteasome inhibitor, and second-generation proteasome inhibitors in leukemia treatment from a preclinical and clinical perspective. Potential markers for proteasome inhibitor sensitivity and/or resistance emerging from leukemia cell line models and clinical sample studies will be discussed focusing on the role of immunoproteasome and constitutive proteasome (subunit) expression, *PSMB5* mutations, and alternative mechanisms of overcoming proteolytic stress.

## Introduction

Hematological malignancies comprise of many subgroups including chronic and acute leukemia, lymphoma, and multiple myeloma (MM). In this review, we focus exclusively on acute leukemia, which can be divided into two major subgroups: acute lymphoblastic leukemia (ALL) and acute myeloid leukemia (AML). In children, the majority of leukemia cases are ALL [1], while AML is more prevalent in adults with leukemia [2]. With a 5-year overall survival (OS) of 83–94% [3], the prognosis of pediatric ALL is considerably better as compared to adults (5-year OS 15–35%, depending on age) [4]. A similar difference in prognosis between children and adults is seen in AML with a 5-year OS of, respectively, 65–70% and 10–45% (depending on age) [5]. The main reasons for treatment failure in both children and adults are intrinsic or acquired drug resistance in a subset of leukemia cells that are responsible for refractory disease or the development of relapse, which have a dismal prognosis. Since the (emergence of) drug resistance is one of the limiting factors that impacts long-term efficacy of anti-leukemic drugs, the search for new anti-leukemic drugs with novel mechanisms of action is an ongoing challenge.

**Fig. 1:**
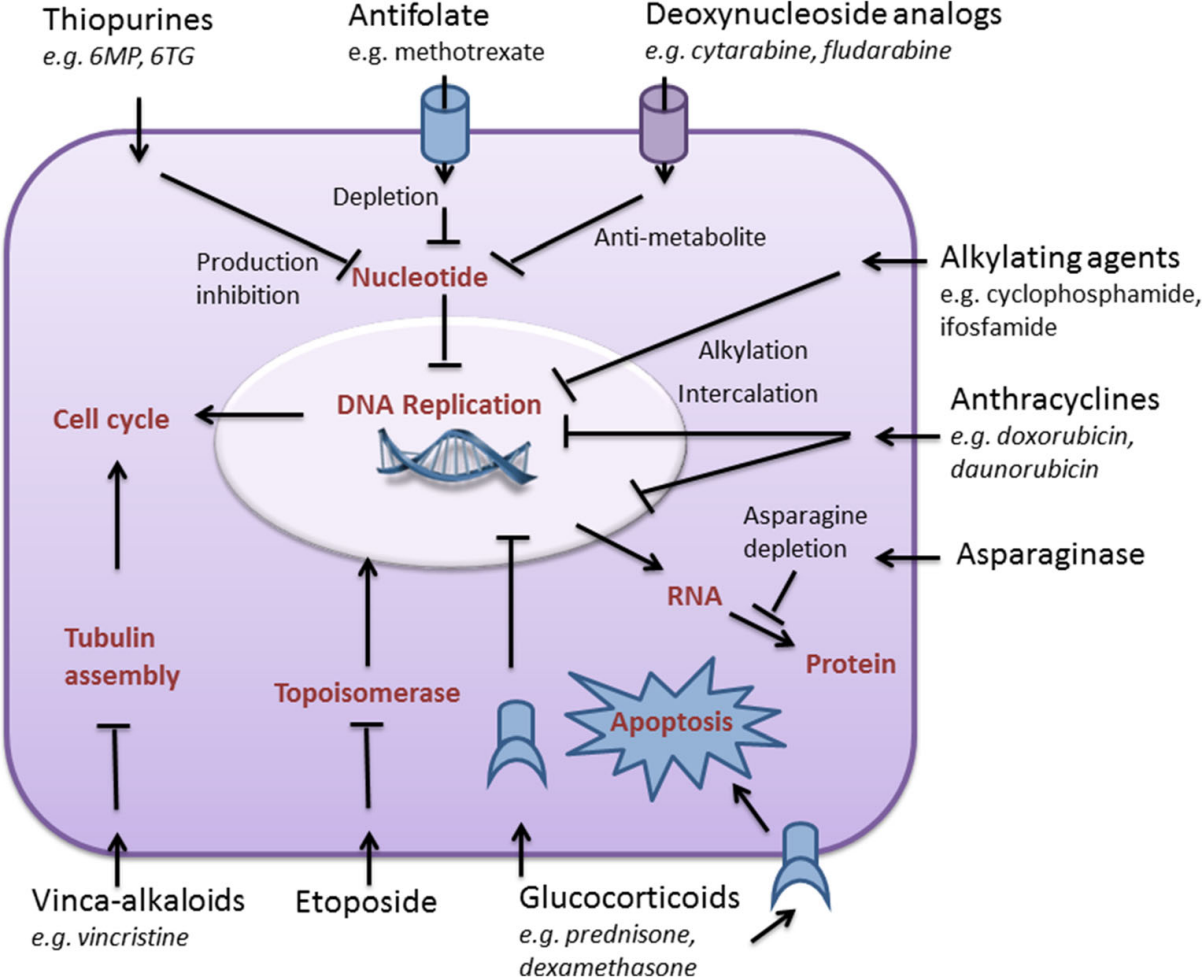
Overview of cytotoxicity mechanism of chemotherapy drugs commonly used in acute leukemia treatment

Most anti-leukemic drugs are targeted against DNA replication to interfere with abundant cell proliferation (Fig. 1). For leukemia cells to expand, they also rely on a very high protein turnover. In normal cells, with normal chromosome numbers and normal protein balance, protein homeostasis is maintained mainly by the ubiquitin-proteasome system (UPS) [6]. Besides rapid cell growth, leukemia cells also feature many chromosomal and molecular aberrations, including chromosomal translocations (e.g*.*, t(8:21), Inv(16)), hypo- and hyper-diploidy, activating mutations (e.g*.*, *FLT3/ITD*, *cKIT*), and splicing defects, the latter leading to many different protein isoforms [7, 8]. Together, this leads to an aberrant protein expression, which imposes an inherent heavy burden on the UPS. These considerations set the stage for therapeutic interventions of UPS-targeting with proteasome inhibitors (PIs) [9], of which bortezomib (BTZ) as the first prototypical PI proved successful in therapy-refractory multiple myeloma (MM) [10]. Currently, first line treatment of MM includes BTZ and extends to clinical evaluations of next-generation PIs. Updates of PI treatment in MM have been subject of several recent dedicated reviews [6, 11, 12].

This review will primarily focus on the application of PIs in acute leukemia, in particular refractory disease. Since many novel drugs with different mechanisms of action are currently available, it is crucial to select those patients for certain PI who will benefit from the treatment. Therefore, it is of clinical relevance to understand the mechanism of action of PIs in leukemia and identify parameters that can help to define (non)-responsiveness to PIs. To this end, this review provides a comprehensive overview on the molecular mechanisms of action and resistance to PIs in leukemia as well as current applications of PIs in clinical trials in leukemia patients.

## Proteasome inhibitors in leukemia

**Fig. 2:**
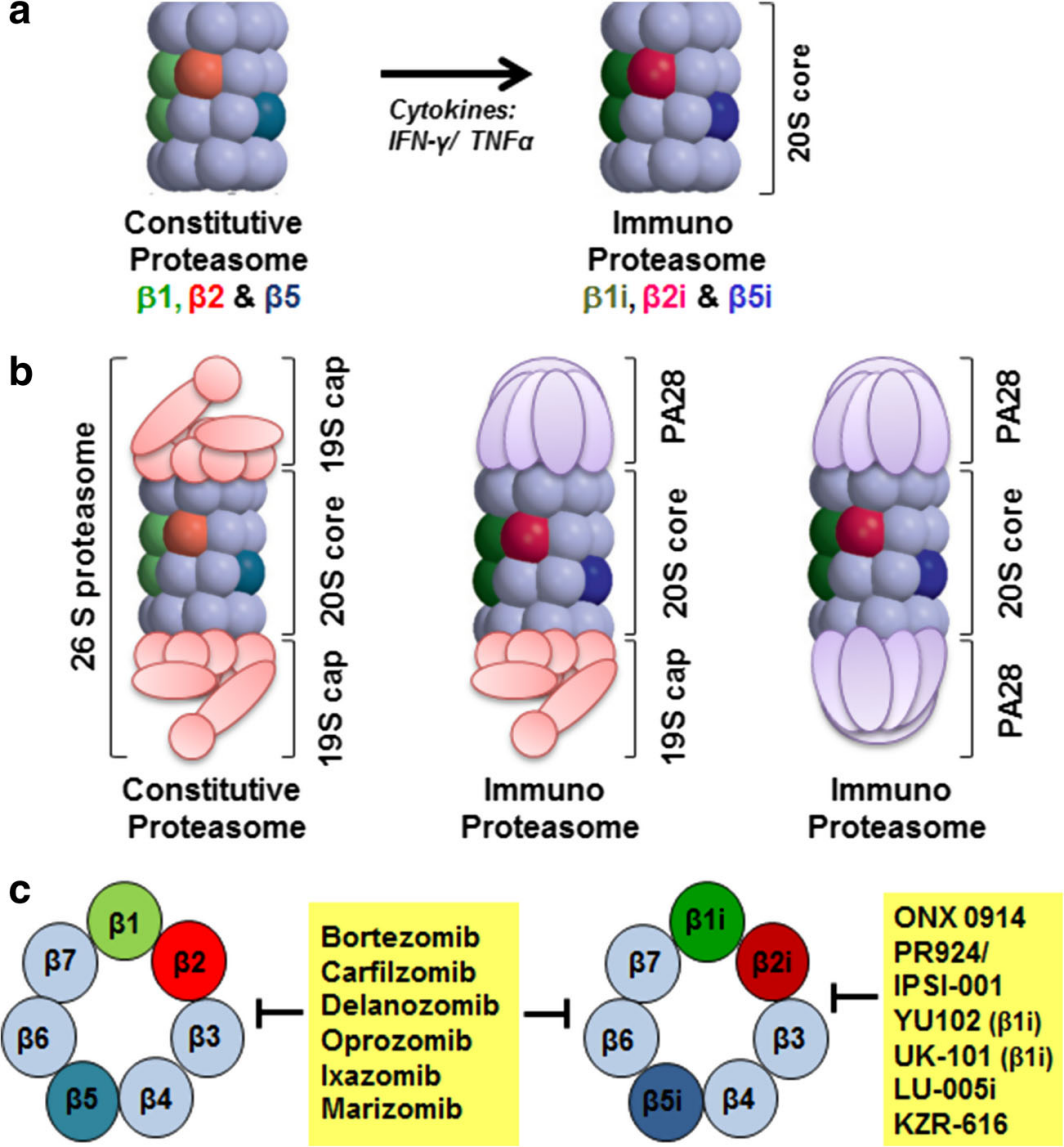
Subunit composition and inhibitors targeting of constitutive and immunoproteasomes. **a** 20S core proteasome and **b** fully assembled immunoproteasome with various cap proteins. **c** Clinically active and experimental inhibitors of constitutive- and/or immunoproteasome. *Adapted from Verbrugge* et al. *2015* [15]

In the context of hematological cells, it is of importance to recognize that the proteasome composition is highly skewed for > 70% towards immunoproteasomes (iP) over constitutive proteasomes (cP) [13, 14]. The three catalytically active β-subunits (β1, β2, and β5) of the constitutive proteasome and the immunoproteasome counterparts β1i, β2i, and β5i harbor caspase-like, trypsin-like, and chymotrypsin-like proteolytic activity, respectively (Fig. 2).

iP expression is markedly induced upon stimulation by inflammatory cytokines such as IFNγ and TNFα [16] (Fig. 2a, b). One of the primary functions of iP is to broaden the spectrum of generating antigenic peptides for presentation on MHC class I molecules [15, 17], but also additional functions for iPs have been defined, e.g., clearance of polyubiquitinated protein aggregates emerging under inflammatory oxidative stress conditions [15, 18–23] (Fig. 2c). Given the abundance of iP in leukemia cells, selective targeting of iP is an attractive treatment option [24].

BTZ and next-generation PIs [25, 26] have been evaluated in preclinical and clinical studies as potential anti-leukemia drugs. An overview of their properties is provided in Table 1. BTZ is a reversible PI primarily targeting the β5 catalytic active subunit of the proteasome. Next-generation PIs differ from BTZ by being irreversible inhibitors (e.g., carfilzomib (CFZ)), favoring oral administration (e.g., ixazomib (IXA)), attenuating hematological and neurological side effects and overcoming BTZ-associated resistance modalities [43–45]. Moreover, these next-generation PIs display selectivity for cP and iP, and subunits other than β5 [27, 28, 46, 47]. Initial *ex vivo* activity studies of BTZ and next-generation inhibitors of cP (CFZ, ONX-0912) and iP (ONX-0914) revealed considerable inter-patient variabilities but overall greater potency in ALL than AML cells [48]. Moreover, BTZ, CFZ, and ONX-0912 were 3–10-fold more potent than the iP inhibitor ONX-914. Interestingly, this study also showed that a higher ratio of immunoproteasome over constitutive proteasome levels in leukemia cells was a good indicator for PI sensitivity. An overview of selected preclinical studies of several PIs in ALL and AML cell lines and primary cells is summarized in Table 2. Overall, these data show the relevance of preclinical studies to unravel the individual specific mechanisms of action of PIs related to apoptosis induction. In addition, these data reveal promising combination strategies for improvement of successful PI therapy.

**Table 1:** Overview of current proteasome inhibitors

Class	Compounds	Binding to proteasome	Specificity and mechanisms
Peptide aldehydes	MG-132, ALLnL, ALLnM, LLnV, PSI	Reversible	Interact with the catalytic threonine residue of the proteasome.
Peptide boronates	Bortezomib, MG-262, PS273 CEP-18770 (delanzomib) MLN9708/MLN2238 (ixazomib citrate/ixazomib)	Reversible	Selective proteasome inhibitors. Interact with the catalytic threonine residue of the proteasome.
Peptide vinyl sulfones	NLVS, YLVS	Irreversible	Interact with β-subunits of the proteasome.
Peptide epoxyketones	Dihydroeponemycin Epoxomycin PR-171 (carfilzomib) PR-047 (ONX 0912, oprozomib)	Irreversible	Selective proteasome inhibitors. Bind specifically to β5-subunit of the proteasome.
	PR-957 (ONX 0914) PR-924		Selective immune proteasome inhibitors. Bind to immune β-subunits of the proteasome.
β-Lactones	Lactacystin	Irreversible	Relatively specific but weak proteasome inhibitors. Binds to β-subunits of the proteasome.
	NPI-0052 (marizomib)	Irreversible	Binds to β-subunits of the proteasome.

Abbreviations: *MG-132* Carbobenzoxy-L-leucyl-L-leucyl-leucinal, *ALLnL* N-acetyl-L-leucyl-L-leucyl-L-norleucinal, *ALLnM* N-acetyl-L-leucyl-L-leucyl-Lmethioninal, *LLnV* N-Carbobenzoxy-L-leucyl-L-norvalinal, *PSI* N-carbobenzoxy-L-isoleucyl-L-γ-t-butyl-L-glutamyl-L-alanyl-L-leucinal, Leu-Leu-vinyl sulfone, *MG-262*N-benzyloxycarbonyl-L-leucyl-L-leucyl-L-leucyl boronic acid. See for details [13, 27–42]

**Table 2:** Selection of preclinical studies of proteasome inhibitors in leukemia

Proteasome inhibitors	Leukemic cells	Study results and mechanisms involved	Refs.
Several	AML cell line HL60	Induction of apoptosis. Increase of p27^Kip1^. Activation of cysteine proteases.	[49]
PSI	CML, AML, and ALL cell lines	Induction of apoptosis in all cell lines. Enhanced taxol and cisplatinum cytotoxicity. PSI was more active on leukemic than on normal CD34^+^ bone marrow progenitors.	[50]
Lactacystin	AML cell line U937	Lactacystin combined with PKC activator bryostatin enhanced apoptosis.	[51]
Lactacystin, MG-132	Primary CLL cells	Induction of apoptosis in both GC sensitive and resistant cells. Activation of cysteine proteases. Apoptosis is blocked by caspase antagonist zVADfmk. Inhibition of NF-κB.	[52]
MG-132, LLnL, lactacystin	AML and ALL cell lines, primary AML cells	Synergistic interactions between PI and cyclin-dependent kinase inhibitors flavopiridol and roscovitine. Downregulation of XIAP, p21^CIP1^, and Mcl-1.	[53]
Bortezomib	Primary CLL cells	Induction of apoptosis associated with release of SMAC and cytochrome c.	[54]
Bortezomib	CML, AML, and ALL cell lines	Synergistic with flavopiridol. Blockade of the IκB/NF-κB pathway. Activation of the SAPK/JNK cascade. Reduction in activity of STAT3 and STAT5.	[55]
Bortezomib	Primary CLL cells	Dose-dependent cytotoxicity of bortezomib. Additive effect with purine nucleoside analogues cladribine and fludaribine. CLL cells more sensitive than normal lymphocytes.	[56]
Bortezomib	AML and ALL cell lines, primary pediatric AML and ALL cells	Lymphoblastoid, CML and AML cell lines. Bortezomib induced apoptosis and acted at least additive with dexamethasone, vincristine, asparaginase, cytarabine, doxorubicin, geldanamycin, HA14.1, and trichostatin A.	[57]
Bortezomib	AML cell lines	Synergistic with tipifarnib. The combination overcomes cell adhesion-mediated drug resistance.	[58]
Bortezomib	Pediatric ALL xenocraft model	*In vitro* and *in vivo* activity of bortezomib against primary pediatric ALL cells in a xenocraft mouse model.	[59]
Bortezomib, PSI	CML and AML cell lines	PSI enhanced toxicity of daunoblastin, taxol, cisplatinum, and bortezomib. PSI and bortezomib suppressed clonogenic potential of AML and CML more than that of normal bone marrow (NBM) progenitors. Bortezomib inhibited the clonogenic potential of CML and NBM more effectively.	[60]
Carfizomib	Primary AML and ALL cells	Inhibits proliferation and induces apoptosis AML, inhibits proliferation in ALL.	[61]
Carfilzomib, bortezomib	AML cell lines and primary AML cells	Synergistic effect on proteotoxic stress together with the protease inhibitors ritonavir, nelfinavir, saquinavir, and lopinavir.	[62]
Carfilzomib, bortezomib	ALL cell lines *in vitro* and in xenograft model	Proteasome inhibitors evoke latent tumor suppression programs in pro-B MLL leukemia through MLL-AF4.	[63]
Carfilzomib	MM, AML, Burkitt’s lymphoma cell lines	Induces proapoptotic sequelae, including proteasome substrate accumulation, Noxa and caspase 3/7 induction, and phospho-eIF2α suppression.	[13]
Marizomib	ALL, AML, and CML cell lines and in xenograft model	Induces caspase-8 and ROS-dependent apoptosis alone and in combination with HDAC inhibitors.	[64, 65]
Marizomib, bortezomib	AML and ALL cell lines	Anti-leukemic activity, synergistic in combination with bortezomib.	[31]
ONX 0914	AML and ALL cell lines	Growth inhibition, proteasome inhibitor-induced apoptosis, activation of PARP cleavage and accumulation of polyubiquitinated proteins.	[16]
PR-924	AML and ALL cell lines	Growth inhibition, immune proteasome inhibition, apoptosis, activation of PARP cleavage.	[29]
Ixazomib	Primary CLL cells	Annexin-V staining, PARP1 and caspase-3 cleavage and an increase in mitochondrial membrane permeability, apoptosis was only partially blocked by the pan-caspase inhibitor z-VAD-fmk.	[66]

Updated from Franke et al. [67]

Abbreviations: *PSI* N-carbobenzoxy-L-isoleucyl-L-γ-t-butyl-L-glutamyl-L-alanyl-L-leucinal, *LLnV* N-Carbobenzoxy-L-leucyl-L-norvalinal, *LLnL* N-acetylleucylleucylnorleucinal, *MG-132* Carbobenzoxy-L-leucyl-L-leucyl-leucinal, *GC* glucocorticoid, *PKC* protein kinase C

## Markers for PI (BTZ) sensitivity/resistance in leukemia cell lines

**Fig. 3:**
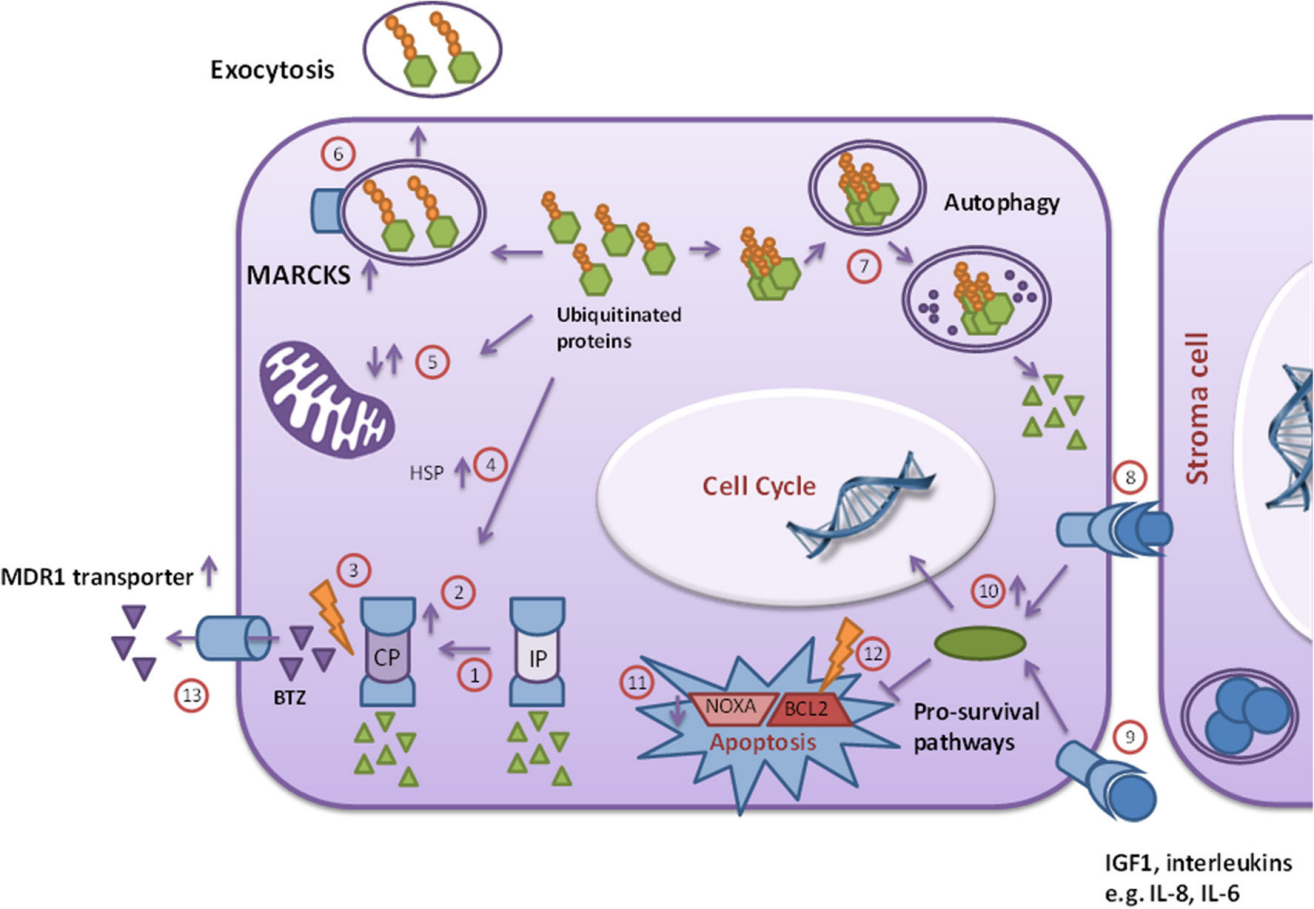
Overview of known molecular mechanisms involved in BTZ resistance. **a** Proteasome related resistance: relative down regulation of immunoproteasome as compared to constitutive proteasome (1) together with absolute upregulation of the constitutive proteasome (2) and mutation in the β5 subunit of the proteasome (3). **b** Alternative stress handling: upregulation of heat shock proteins (4) or changes in redox metabolism (5) which can prevent oxidative stress. Alternative handling ubiquitinated protein: exocytosis of ubiquitinated proteins in MARCKS-associated vesicles (6), and protein degradation through autophagy (7). **c** Activation pro-survival signaling: intrinsic activation of pro-survival pathways, e.g., AKT, NFκB, or MET (8) or through stimulation by direct interaction with stromal cells (9) or indirectly through soluble growth factors or interleukins (10). **d** Decreased apoptosis: downregulation (11) or mutation (12) of pro-apoptotic proteins. Finally, **e**, extrusion of BTZ *via* multidrug resistance efflux pump MDR1/Pgp (minor effect, more pronounced in CFZ resistance) (13). CP, constitutive proteasome; IP, immunoproteasome; MDR1, multidrug resistance protein 1; HSP, heat shock proteins; IGF1, insulin-like growth factor 1; IL, interleukin

As with any new treatment strategy, selection of patients who will benefit from the treatment is essential. With respect to PIs, studies with leukemia cell lines can help to define markers for response, long-term efficacy, and emergence of resistance to PIs. Resistance mechanisms often reveal critical processes such as targeted and compensatory mechanisms that leukemia cells harbor to overcome the effects of PIs. A large number of studies have followed the approach of exposing leukemia cells to stepwise increasing concentrations of PIs (mostly BTZ) and characterize cells with acquired resistance [68–71]. Figure 3 depicts an overview of molecular mechanisms of resistance in PI/BTZ-resistant leukemia cell lines [44, 46, 72]. The most common mechanisms are discussed below.

### Upregulation of proteasomal subunits

Upregulation of the primary target is a well-known response of cells exposed to drugs. Likewise, upregulation of proteasomal subunits, and the β5 subunit in particular, is frequently observed in BTZ-resistant leukemia cell lines (reviewed in [44, 46]) indicating its role in BTZ resistance. Consistently, CGH analysis of BTZ-resistant cell lines revealed amplification of the *PSMB5* gene with variability in size and extent of the amplification of chromosome 6 [73]. Remarkably, immunoproteasome subunit levels are often found to be decreased in BTZ-resistant leukemia cell lines [16, 68, 71]. The resulting decrease in immuno- / constitutive proteasome ratio and alterations in subunit composition has been linked to a diminished BTZ sensitivity [74, 75]. Interferon gamma (IFNγ) is an efficient inducer of immunoproteasome expression and partly restored BTZ sensitivity in BTZ-resistant leukemia cells [16]. Beyond BTZ-resistant cells, IFNγ also enhanced BTZ sensitivity in a panel of B cell lines by 50% [76]. Together, these data underscore the importance of immuno- and constitutive proteasome subunit composition in BTZ sensitivity and resistance in leukemia cells.

### *PSMB5* mutations

**Fig. 4:**
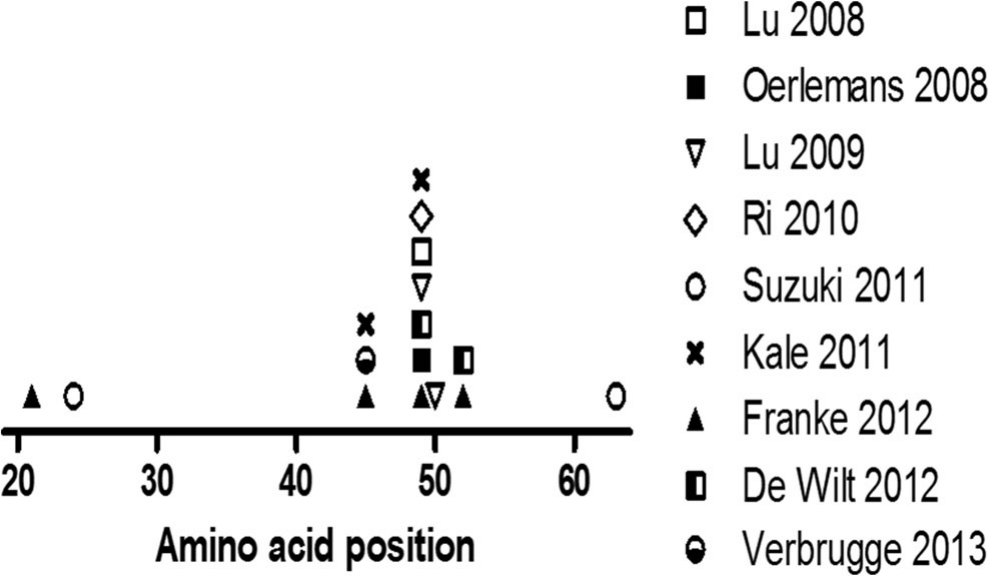
Clustering of *PSMB5* mutations in several BTZ-resistant *in vitro* model systems [68–71, 77–81]

Molecular analysis of the proteasome subunits of PI-resistant leukemia cell lines revealed several mutations in exon 2 of the *PSBM5* gene encoding the highly conserved binding pocket region for PIs within the β5 subunit of the proteasome. This highly conserved region appears to be a mutation “hot spot” when cells are exposed to BTZ for a prolonged period. Figure 4 shows an overview of *PSMB5* mutations in PI-resistant cell lines, including non-hematological malignancies.

All mutations result in amino acid alteration in, or in the close vicinity of, the PI binding pocket [82]. In fact, except for the A247G mutation which introduces a Thr21Ala substitution, all mutations result in amino acid alterations in the S1 pocket of the β5 subunit with Ala49 being the most affected amino acid. This specificity pocket is a highly conserved part of the subunit and responsible for recognizing the peptide bond of the substrate. It is also the site that has to be cleaved and determines the specificity as well as facilitates the binding of the P1 side chain of BTZ [82]. *In silico* analysis provided evidence for hindered binding of BTZ by the majority of the mutations, including the Thr21Ala amino acid alteration positioned outside of the S1 pocket but within the BTZ binding pocket [82]. The only exceptions were the Met45Ile and Met45Val substitutions, which do not directly interact with BTZ. However, Met45 is known to contribute to the specificity of the S1 pocket and upon binding, Met45 undergoes a conformational change and shifts the direction of its side chain towards Cys52 vicinity. Alterations in this amino acid might therefore hinder this conformational change and contribute as well to decreased BTZ binding. Docking the LLVY-AMC substrate *in silico* in the crystal structure of the β5 subunit supported the hypothesis of decreased binding of the substrate in the mutated binding pocket.

There are two strong indications that *PSMB5* mutations are related to PI resistance. First, from marine biology studies, characterization of proteasome subunits of the PI Salinosporamide A (NPI-0052, Marizomib) producing actinobacterium *Salinispora tropica* revealed the same Ala49Val and Met45Phe mutations in the β5 subunit homologue as in BTZ-resistant leukemia cells. In *S. tropica*, these mutations conferred “self-resistance” to Salinosporamide A [80]. Second, acquired resistance to the immunoproteasome inhibitor in PR-924 was not associated with any mutation(s) in immunoproteasome subunits, but rather provoked *PSMB5* mutations, i.e., Met45Ile, Ala49Thr, and Met45Val [29]. Taken together, inhibition of the β5 subunit is essential for the anti-leukemia effect of PIs and *PSMB5* mutations emerging after prolonged PI treatment attenuate the inhibitory potency and confer resistance in leukemia cells. *PSMB5* mutations have thus far not been identified in patients receiving PI therapy and it has been challenged whether these mutations hold clinical relevance for PI resistance. However, until now, longstanding treatment with the first-generation PI was hardly applied in clinical practice. This may change with the introduction of second-generation and oral PI’s allowing for long-term treatment.

### Alternative protein disposal

Gene expression profiling of BTZ-resistant leukemia cells identified myristoylated alanine-rich C-kinase substrate (MARCKS) as being highly upregulated. MARCKS is an 80-kDa protein that is involved in multiple exocytosis pathways (reviewed in [83]). In fact, in BTZ-resistant leukemia cells, MARCKs protein co-localized with intracellular vesicles that contained polyubiquitinated proteins and which were formed upon exposing cells to increasing concentrations of BTZ. The ubiquitin-containing vesicles have been described before [84], and in co-cultures, it was shown that after extrusion of the vesicles, they were taken up in proteasome-proficient acceptor cells [73]. MARCKs may thus contribute to BTZ resistance by facilitating exocytosis-mediated extrusion of polyubiquitinated proteins to overcome proteolytic stress imposed by BTZ. MARCKs upregulation is not restricted to BTZ-resistant leukemia cells, but was also observed in leukemia cells with acquired resistance to second-generation PI, e.g., Salinosporamide A (Marizomib) and the immunoproteasome inhibitor PR924.

Another alternative protein disposal that is often related to drug resistance is autophagy. This process has been linked to BTZ resistance in several tumor models [85–91], although it was not indicated in MARCKs overexpressing PI-resistant leukemia cells [73]. Inhibition of autophagy through different mechanisms including calpain inhibitor [86], downregulation of heat shock protein B8 (HSPB8) [87], B cell lymphoma 2-interacting mediator of cell death (BIM) upregulation [90], tipifarnib [85], or (hydroxy)chloroquine [92, 93] increased BTZ sensitivity in a MM model. In addition, histone deacetylase (HDAC) inhibitors can block autophagy by disrupting aggresome formation, the process that precedes autophagy [94, 95]. Interestingly, the E3 ligase, TRAF6, links the NFkB pathway to autophagy [96] and it was shown that BTZ-resistant AML cells were sensitized by downregulating TRAF 6 [97]. Despite the potential impact of autophagy on BTZ resistance, the extent of involvement of this process in leukemia cells needs further exploration.

### Activation of pro-survival pathways

Analogous to resistance to many anti-leukemia drugs, PI resistance has also been associated with the activation of pro-survival pathways. The most extensively described is the association with the NFκB survival pathway (for AML reviewed in [98]). As PI result in the stability of the inhibitor of NFκB (IκB), this survival pathway is inhibited by exposure to PI. Intrinsically resistant leukemia cells (e.g., stem cells) have a constitutively activated NFκB pathway and the combination of BTZ with NFκB inhibition by the IKK inhibitor BMS-345541, enhanced the kill of AML stem cells [99]. Although the interaction between NFκB and PI resistance is clearly established for MM and other tumor types [100–104], the data of relevance for leukemia is limited. Besides NFκB, the pro-survival pathways *via* AKT/mTOR [105–107] and insulin-like growth factor 1 (IGF-1) [108–110] have also been described to confer BTZ resistance and may affect the interaction of leukemia cells with (stromal) cells in their micro-environment.

### X-box binding protein 1 (XBP1)

X-box binding protein 1 (XBP1) is a transcription factor involved in the unfolded protein response. In addition, it is important for the differentiation of plasma cells. In this respect, XBP1 expression has been linked to BTZ resistance [111] and survival [112] in MM. In leukemia, this pathway may also be related to PI resistance; however, data are currently lacking. Due to the role of XBP1 in B cell differentiation, this factor may be particularly relevant for B-ALL and warrants further study.

### Drug efflux transporters

Drug efflux transporters of the ATP-binding cassette protein family have been explored for their role in PI resistance. P-glycoprotein (Pgp, ABCB1), as one of its main representatives only marginally contributes to BTZ efflux [68, 113], but is of relevance for second-generation PIs such as CFZ and the immunoproteasome inhibitor ONX-0914 as these PIs are *bona fide* substrates for Pgp [114, 115]. Notably, BTZ can downregulate Pgp expression and this manner indirectly attenuate drug resistance [116]. There is no evidence that BTZ and other second-generation PIs are substrates for other drug efflux transporters, e.g., multidrug resistance-associated protein 1–5 (MRP1–5, ABCC1–5) or breast cancer resistance protein (BCRP, ABCG2); hence, a role in PI resistance is not anticipated [114].

## Markers for PI (BTZ) sensitivity/resistance in primary leukemia samples

Although *in vitro* models are valuable tools to identify possible mechanisms of BTZ resistance, assessment of the relevance for the clinic requires validation in *ex vivo* studies using primary patient samples. Preferably, this is studied in add-on studies of clinical trials that include BTZ in the treatment protocol. The potential role of three markers for PI (BTZ) sensitivity and/or resistance in leukemia samples is discussed below.

### Proteasome levels and subunit composition

Add-on studies of clinical trials in leukemia are limited and most data for PIs are obtained from MM studies. These studies demonstrated besides proteasome expression levels, the proteasome subunit composition is important for response [117, 118]. Although upregulation of the proteasome was not related to resistance in mantle cell lymphoma (MCL) patients [119], studies in leukemia indicated a possible correlation between higher 20S protein expression and BTZ sensitivity [120]. In particular, studies by Niewerth et al. (2013) showed that lower β5 subunit expression correlated with increased *ex vivo* sensitivity for proteasome inhibitors in pediatric AML and ALL samples. In addition, the sensitivity for BTZ in AML cells inversely correlated with the ratios between immunoproteasome subunits over constitutive subunits, specifically β1i/β1 and β2i/β2, and a trend for β5i/β5. ALL cells showed higher sensitivity to BTZ as compared to AML cells. Although for ALL no significant correlations were revealed with BTZ sensitivity, they featured a higher β2i/β2 ratio and trends of a higher β1i/β1 ratio as compared to AML samples. Together, these data support the notion that a relative high immuno- / constitutive proteasome ratio promotes BTZ sensitivity. These data were confirmed in AML and ALL samples obtained from two pediatric clinical COG trials (AAML07P1 and AALL07P1) in which BTZ treatment was incorporated [14, 48]. After further validation in larger studies, assessment of immunoproteasome over constitutive proteasome ratios may be used as biomarkers of response to PIs.

### PSMB5 mutations

*PSMB5* mutations observed in BTZ-resistant hematological cell lines were as yet not identified in clinical samples. The mutations found in the cell line models do not represent SNPs found in the general population. Wang et al. sequenced the PSMB5 gene in a large cohort of healthy persons and 61 MM patients after BTZ treatment [121]. No SNPs were found in the exon 2 of the *PSMB5* gene neither in the general population nor in MM patients. Of interest, polymorphisms that influenced *PSMB5* gene expression were observed, but these did not correlate with BTZ response. It should be taken into account that sequence analysis in one third of the MM patients was performed only on whole blood and not on isolated malignant plasma cells, which may have influenced the sensitivity of the analysis. In addition, it was not stated how many resistant patients were included. More recently, Lichter et al. sequenced the *PSMB5* gene in blood samples of MM patients included in the APEX trial in which patients were treated with either BTZ or dexamethasone [122]. No *PSMB5* mutations were identified in this group. Although sample size of this group was limited, the data suggest that *PSMB5* mutations do not represent a leading cause of acquired BTZ resistance in investigated MM protocols. Whether this holds true for leukemia or MM with long-term BTZ maintenance therapy is yet to be determined. Lastly, as a preliminary account, Barrio et al. (2016) reported *PSMB5* mutations in subclones of CD138+ cells of a single MM patient after a therapy [123]. Since the subclonal frequencies were low, this poses analytical challenges to detect these subclones. Also in leukemia, it is established that there is substantial oligoclonality in mutational status of cells within the leukemia. The cells with the specific mutations tendering growth advantage are probably selected to grow out and develop a (drug resistant) relapse [124], so also in leukemia patients subclonal analysis may reveal additional mutations.

### MARCKS

Upregulation of MARCKs protein expression emerged as a marker for BTZ- and second-generation PI-resistant leukemia cell lines [73]. To test whether this upregulation might be a prognostic marker for clinical BTZ resistance, MARCKs expression was examined in 44 primary ALL patient samples obtained from the clinical COG trial AALL07P1 using combination chemotherapy including BTZ. A trend (*p* = 0.07) was seen in the inverse correlation between MARCKS expression and clinical response. Since the samples were obtained in the setting of a clinical trial using combination therapy, a direct correlation between BTZ response and MARCKS expression cannot be made. However, these findings are consistent with data from Micallef et al. who showed MARCKS protein upregulation in a small group of BTZ-resistant MM patients [125] and Yang et al. who identified MARCKS upregulation as a resistance marker in primary MM samples [126]. These studies encourage a prospective validation of the possible prognostic role of MARCKS in leukemia.

## Clinical trials with proteasome inhibitors in leukemia

The efficacy and safety of PIs in MM have already been extensively reviewed [127]. For leukemia, an overview of phase I/II clinical studies of PI as single agent and combination therapy in adult and pediatric leukemia is presented in Table 3. An overview of ongoing phase II/III clinical trials with BTZ (combination) therapy involving pediatric leukemia patients and adult leukemia is shown in Table 4 and Table 5, respectively. In addition, ongoing clinical trials with second-generation PIs (mainly CFZ and IXA) are depicted in Table 6. The outcome for clinical efficacy included stable disease, progressive disease, complete/partial remission, and mortality. In all studies, infections and neutropenia were common adverse drug reactions (ADRs). Neurologic ADRs were also common in all studies with BTZ, including neuropathy. Interestingly, BTZ might also have some protective effects as well, as it has been reported to prevent muscle wasting [148], which can be induced by cancer cachexia [149] but this has not yet been reported in a clinical setting. Two studies were conducted with CFZ [143, 144] and less ADRs were found in these studies including the absence of neurologic ADRs. Moreover, no dose-limiting toxicities (DLT) were found indicating a better safety profile. Importantly, the clinical response to CFZ in these studies was also better than reported for BTZ treatment.

**Table 3:** Published clinical studies of proteasome inhibitors in leukemia

Study drugs	Cohort	Number	Phase	Study results and mechanisms involved	Refs.
BTZ	Several hematologic malignancies	27	I	Bortezomib was given twice weekly for 4 weeks every 6 weeks. The MTD was 1.04 mg/m^2^. CR in 1 MM patient. PR in 1 patient with MCL and 1 with FL.	[128]
BTZ	Refractory or relapsed acute leukemia	15	I	Bortezomib was given twice weekly for 4 weeks every 6 weeks. The MTD was 1.25 mg/m^2^. No ≥grade 3 toxicities. 5 patients showed hematological improvement. No CR achieved.	[129]
BTZ, PegLD	AML, MM, and NHL	42	I	Bortezomib was given on days 1, 4, 8, and 11 and PedLD on day 4. MTD of BTZ 1.3 mg/m^2^. No significant pharmacokinetic and pharmacodynamic interactions between bortezomib and PegLD. 16 of 22 MM patients achieved CR, near-CR or PR. 1 CR and 1 PR in NHL patients. 2 of 2 AML patients achieved a PR.	[130]
BTZ	Recurrent childhood ALL, AML, blastic phase CML, M3	12	I	Bortezomib was administered twice weekly for 2 weeks followed by a 1-week rest. MTD of bortezomib was 1.3 mg/m^2^/dose. 5 patients were fully evaluable. DLT’s occurred in 2 patients at the 1.7 mg/m^2^ dose level. No OR achieved.	[131]
BTZ, IDA, AraC	AML	31	I	Addition of BTZ to AML induction chemotherapy. Bortezomib added on days 1, 4, 8, and 11. 19 CR, 3 CRp, 2 PR and 7 no response. BTZ was well-tolerated up to 1.5 mg/m^2^.	[132]
BTZ, VCR, DEX, PegAspa, DOX	Recurrent childhood ALL	10	I	Combination of bortezomib (1.3 mg/m^2^) with ALL induction therapy is active with acceptable toxicity. 6 patients achieved CR.	[133]
BTZ, VCR, DEX, PegAspa, DOX	Recurrent childhood ALL	22	II	14 patients achieved CR, and 2 achieved CRp, 3 patients died from bacterial infections, 2 of 2 included T cell ALL patients did not respond.	[134]
BTZ, tipifarnib	Relapsed or refractory ALL(26) or AML (1)	27	I	Combination well tolerated. 2 patients achieved CRp and 5 SD.	[135]
BTZ, DNR, AraC	AML (age > 65)	95	I/II	Combination was tolerated. 62 patients achieved CR and 4 patients CRp.	[136]
BTZ, 17-AAG	Relapsed or refractory AML	11	I	The combination of 17-AAG and BTZ led to toxicity without measurable response in patients with relapsed or refractory AML.	[137]
BTZ, DAC	Poor-risk AML	19	I	Combination was tolerable and active in this cohort of AML patients; 7 of 19 patients had CR or CRi. 5 of 10 patients > 65 years had CR.	[138]
BTZ, LEN	14 MDS/CMML 9 AML	23	I	MTD of BTZ 1.3 mg/m^2^ was tolerable in this regimen. Responses were seen in patients with MDS and AML. Two fatal infections occurred.	[139]
BTZ, IDA	Relapsed AML (7) or AML > 60 years (13)	20	I	4 patients achieved complete remission. 1 treatment-related death. Overall the combination was well tolerated.	[140]
BTZ, AZA	Relapsed or refractory AML	23	I	Dose of 1.3 mg/m^2^ BTZ was reached without dose-limiting toxicities. 5 out of 23 patients achieved CR.	[141]
BTZ, MIDO *vs* BTZ, MIDO, DHAD, etoposide, AraC	Relapsed/refractory AML	21	I	56.5% CR rate and 82.5% overall response rate (CR + CR with incomplete neutrophil or platelet count recovery). Combination is active but is associated with expected drug-related toxicities. DLTs were peripheral neuropathy, decrease in ejection fraction and diarrhea.	[142]
CFZ + dexamethasone	Refractory or relapsed acute leukemia	18	I	CFZ was given twice weekly for 4 weeks with a maximal of 6 cycles. Prior to CFZ dexamethasone was given. The MTD was not established, because no DLTs were observed (36–46 mg/m^2^). PR in 2/10 patients and 4/10 SD.	[143]
CFZ + dexamethasone	Previously treated patient with CLL or SLL	19	I	CFZ was given twice weekly for 3 weeks in a 28-day cycle. Prior to CFZ dexamethasone was given. No DLTs observed and all available patients for evaluation had SD (including patients with Del(17p13.1) and fludarabine-resistant disease.	[144]
BTZ, cytarabine, idarubicin *vs* BTZ, cytarabine etoposide	Children with relapsed, refractory, or secondary AML	37	II	BTZ, 1 or 1.3 mg/m^2^, was given at days 1, 4, and 8 in combination with idarubicin and cytarabine (arm A) or with etoposide and high dose cytarabine (arm B). Hypokalemia incidence was high, 17%. Four deaths occur, 3 infectious deaths and one from PD. Both arms failed to meet predetermined efficacy thresholds (CRi was not included). Arm A: CR = 21.4%, CRp + CRi = 35.6%, PR = 14.3%. Arm B: CR = 34.8%, CRp + CRi = 13% and one death.	[145]
BTZ	Relapsed/refractory ATL	15	II	BTZ, 1.3 mg/m^2^, was given at days 1, 4, 8, and 11. After stage 1, all patients discontinued treatment (PD = 11, AEs = 3) and the study was terminated because BTZ was not considered promising enough as a single agent. 12 patients had Gr 3/4 drug-related AEs of which 2 Gr3/4 peripheral neuropathy. Overall responses: PR = 1, SD = 5. ORR = 6.7%, PFS = 38 days (8–122).	[146]
BTZ, DEX, DOX *vs* BTZ, DEX, cyclophosphamide	Newly diagnosed primary plasma cell leukemia	39	II	Four alternating cycles of BTZ (1.3 mg/m^2^ on days 1, 4, 8, and 11), DEX plus DOX, or cyclophosphamide was given. 35 patients completed the 4 cycles. ORR = 69%, CR = 10%, VGPR = 26%, PR = 23%. 10 were refractory to the induction phase, and 2 deaths due to sepsis occur. 25 patients underwent HDM/ASCT and 1 a syngeneic allograft. After ASCT: ORR = 92% CR = 34%, VGPR = 38%, PR = 16%, PD = 8%. In the intention-to-treat population, the median PFS = 15.1 months and overall survival = 36.3 months.	[147]

Updated from Franke et al. [67]

Abbreviations: Study outcome: *MTD* maximum tolerated dose, *DLT* dose-limiting toxicities, *CR* complete response, *CRi* incomplete remission, *CRp* CR with incomplete platelet recovery, *PR* partial response, *OR* objective response, *SD* stable disease, *PFS* progression-free survival, *EFS* event-free survival, *OS* overall survival. Malignancies: *MCL* mantle cell lymphoma, *FL* follicular lymphoma, *NHL* non-Hodgkin lymphoma. Drugs: *17-AAG* 17-N-Allylamino-17-Demethoxygeldanamycin, *AraC* cytarabine, *AZA* azacitidine, *BTZ* bortezomib, *CFZ* carfilzomib, *DAC* decitabine, *DEX* dexamethasone, *DHAD* mitoxantrone, *DNR* daunorubicin, *DOX* doxorubicin, *IDA* idarubicin, *LEN* lenalidomide, *PegLD* pegylated liposomal doxorubicin, *PegAspa* pegylated L-asparaginase, *VCR* vincristine

**Table 4:** Ongoing and unpublished clinical trials of bortezomib in acute leukemia which include pediatric patients

Study drugs	Time period	Number	Phase	Cohort	Age	Sponsor	Clinical trial identifier
BTZ + intensive reinduction chemotherapy	Mar 2009 Sept 2014	60	II	Relapsed ALL	1–31	National Cancer Institute (USA)	NCT00873093
BTZ, DEX, VCR, MTX	Sep 2009 Jul 2014	24	II	Relapsed/refractory ALL	0.5–19	Erasmus Medical Center (Rotterdam, The Netherlands)	NTR1881^a^
BTZ, ATO	May 2013 May 2018	30	II	Relapsed acute promyelocytic leukemia (APL)	1–75	Christian Medical College (Vellore, India)	NCT01950611
Standard leukemia chemotherapy ± BTZ	Apr 2014 Feb 2019	1400	III	T cell ALL or stages II–IV T cell lymphoblastic lymphoma	2–30	National Cancer Institute (USA)	NCT02112916
BTZ, SAHA + reinduction chemotherapy	Apr 2015 Apr 2019	30	II	Refractory or relapsed MLL rearranged leukemia	< 21	St Jude Children’s Research Hospital (Memphis, TN, USA)	NTC 02419755
BTZ, PANO + reinduction chemotherapy	Dec 2015 Apr 2019	40	II	Relapsed T cell leukemia or lymphoma	< 21	St Jude Children’s Research Hospital (Memphis, TN, USA)	NCT02518750
BTZ + induction chemotherapy	Oct 2015 Oct 2020	50	I/II	Infant leukemia and lymphoblastic lymphoma	< 1	St Jude Children’s Research Hospital (Memphis, TN, USA)	NCT02553460
BTZ + reinduction chemotherapy	July 2015 Apr 2019	20	II	Refractory or relapsed leukemia and lymphoblastic lymphoma	1–39	Children’s Mercy Hospital (Kansas City)	NCT02535806
BTZ + HR reinduction chemotherapy	Aug 2015 Aug 2018	250	II	High-risk (HR) relapsed ALL	< 18	Charité - Universitätsmedizin (Berlin, Germany)	EudraCT number: 2012–000810-12^a^

Updated from Franke et al. [67]

Abbreviations: Drugs: *ATO* arsenic trioxide, *BTZ* bortezomib, *DEX* dexamethasone, *MTX* methotrexate, *PANO* panobinostat, *SAHA* vorinostat, *VCR* vincristine

^a^Source: www.clinicaltrials.gov and www.skion.nl

**Table 5:** Ongoing and unpublished clinical trials of proteasome inhibitors in adult acute leukemia

Study drugs	Time period	Number	Phase	Cohort	Age	Sponsor	Clinical trial ID
BTZ, DHAD, VP16, AraC	Jan 2006 Sept 2016	55	I/II	Relapsed/refractory acute leukemia	> 18	Thomas Jefferson University (PA, USA)	NCT00410423
BTZ, FLAG, IDA	Apr 2008 Jan 2013	40	I/II	Refractory or relapsed AML	> 18	PETHEMA Foundation	NCT00651781
BTZ, SAHA, SFN	Feb 2010 Sept 2016	38	I/II	Poor-risk AML	> 18	Indiana University (IN, USA)	NCT01534260
BTZ, BEL	May 2010 Feb 2014	24	I	Relapsed/refractory acute leukemia	> 18	Virginia Commonwealth University (VA, USA)	NCT01075425
BTZ, NFV	July 2010 Mar 2013	18	I	Relapsed or progressive advanced hematologic cancer	> 18	Swiss Group for Clinical Cancer Research (Switzerland)	NCT01164709
BTZ, DHAD, VP16, AraC	July 2010 May 2014	34	I	Relapsed/refractory AML	18–70	Case Comprehensive Cancer Center (OH, USA)	NCT01127009
Several drugs in randomization arms ± BTZ	June 2011 June 2017	1250	III	Initial AML	> 29	National Cancer Institute (USA)	NCT01371981
DAC *vs* BTZ, DAC	Nov 2011 June 2015	172	II	AML	> 60	National Cancer Institute (USA)	NCT01420926
BTZ, DOX, PegAspa, VCR, DEX, AraC, MTX	Mar 2013 July 2017	17	II	Relapsed/refractory ALL	> 18	National Cancer Institute (USA)	NCT01769209
BTZ, SFN, DAC	July 2013 Dec 2016	30	I	AML	> 60	National Cancer Institute (USA)	NCT01861314
BTZ, DOX	Mar 2015 Mar 2017	30	II	AML	18–80	University of California, Davis (CA, USA)	NCT01736943
BTZ, LEN	Mar 2015 Aug 2018	24	I	Relapsed AML and MDS after Alllo SCT	> 18	Massachusetts General Hospital (MA, USA)	NCT023121

Updated from Franke et al. [67]

Source: www.clinicaltrials.gov

Abbreviations: Drugs: *17-AAG* 17-N-Allylamino-17-Demethoxygeldanamycin, *AraC* cytarabine, *BEL* belinostat, *BTZ* bortezomib, *DAC* decitabine, *DEX* dexamethasone, *DHAD* mitoxantrone, *DNR* daunorubicin, *DOX* doxorubicin, *IDA* idarubicin, *FLAG* fludarabine, *Ara-C* cytarabine, *G-CSF* granulocyte colony-stimulating factor, *LEN* lenalidomide, *MTX* methotrexate, *NFV* nelvinavir, *PegLD* pegylated liposomal doxorubicin, *PegAspa* pegylated L-asparaginase, *SAHA* vorinostat, *SFN* sorafenib, *VCR* vincristine, *VP16* etoposide

**Table 6:** Ongoing clinical trials of second-generation proteasome inhibitors in acute leukemia

Study drugs	Time period	Number	Phase	Cohort	Age	Sponsor	Clinical trial ID
CFZ	Sept 2010 Jul 2015	18	I	Relapsed/refractory ALL and AML	> 18	Washington University School of Medicine (MO, USA)	NCT01137747
IXA, DHAD, VP16, AraC	May 2014 Nov 2017	30	I	Relapsed/refractory AML	18–70	Case Comprehensive Cancer Center; National Cancer Institute (NCI)	NCT02070458
IXA	Mar 2014 Mar 2016	16	II	Relapsed/refractory AML	> 18	Stanford university/National Cancer Institute (NCI)	NCT02030405
IXA, DHAD, VP16, AraC	Oct 2014 Nov 2018	30	I	Relapsed/refractory AML	18–70	Case Comprehensive Cancer Center (USA)	NCT 02070458
CFZ, DEX, DHAD, PegAspa, VCR	Dec 2014 Jul 2017	39	I/II	Relapsed/refractory AML	< 18	Onyx Therapeutics Inc. (CA, USA)	NCT02303821
CFZ, CYCLO, VP16	Jul 2015 Dec 2017	50	I	Relapsed leukemia and solid tumors	6–29	Phoenix Children’s Hospital (AZ, USA)	NCT 02512926
IXA + induction and consolidation chemotherapy	Nov 2015 Feb 2022	54	I	AML	> 60	Massachusetts General Hospital (MA, USA)	NCT02582359

Updated from Franke et al. [67]

Source: www.clinicaltrials.gov

Abbreviations: Drugs: *AraC* cytarabine, *CFZ* carfilzomib, *CYCLO* cyclophosphamide, *DEX* dexamethasone, *DHAD* mitoxantrone, *IXA* ixazomib, *VCR* vincristine, *VP16* etoposide

## Future perspectives

This overview of results of PI in leukemia reveals that treatment with PI as monotherapy may not give satisfactory clinical responses. To improve the employability of PI in leukemia, several factors are implied to be considered.

### Combination strategies

For leukemia, chemotherapy commonly consists of cocktails of different drugs with different mechanisms of actions and different side effects to exert optimal treatment response with achievable dosages. For novel drugs, it is therefore essential that they can be combined with the most effective drugs currently used. Based on the mechanism of action, glucocorticoids are good candidate drugs to be combined with PI. BTZ and dexamethasone showed synergy in *ex vivo* combination studies in primary pediatric ALL samples [48] clinically BTZ was combined with induction therapy including dexamethasone, vincristine, PEG-asparaginase, and doxorubicin in relapsed pediatric ALL, showing promising CR rates [133, 134]. Currently, several clinical trials are ongoing (Tables 4 and 5), combining BTZ with several chemotherapy protocols which include the standard chemotherapeutics and novel strategies (heat shock protein inhibitors, HDAC inhibitors, and autophagy inhibitors). Based on preclinical data and expected toxicity profiles of the different drugs, the addition of PI to other chemotherapeutics seems a fruitful strategy. In addition, recently the nuclear transport (XPO1) inhibitor selinexor has been combined with BTZ [150] and is currently in a clinical trial for MM (NCT03110562). Whether this is also a relevant strategy for leukemia still has to be established.

### Second-generation proteasome inhibitors

Despite the successful introduction of BTZ, several drawbacks such as resistance and toxic side effects led to development of second-generation proteasome inhibitors which are at several stages of clinical development (Table 6). Due to promising preclinical studies, the irreversible proteasome inhibitor CFZ has advanced rapidly into the clinic for MM as well as leukemia, and is supposed to be a promising alternative for BTZ and might even overcome BTZ resistance.

Recently, an oral formulation resembling BTZ, ixazomib (MLN9708), has emerged into the clinic, with two clinical trials investigating the efficacy of ixazomib in AML (NCT0230405 and NCT 02070458).

PR-957 (ONX 0914) and PR-924 represent members of a new class of proteasome inhibitors being directed specifically against the immunoproteasome [27, 30]. PR-924 demonstrated preclinical efficacy in leukemia and MM [29, 30]. In addition, *ex vivo* cytotoxicity of PR-957 was shown in primary leukemia samples [29]. Since leukemia cells, especially ALL cells, express high levels of immunoproteasome, it might be a good candidate for further clinical development in hematological malignancies.

Development of acquired resistance to the second-generation PIs [29, 31] has to be taken in account as well. Hence, combination therapy is also recommended for the treatment of leukemia with these novel PIs in order to circumvent toxicity and resistance.

### Biomarkers of clinical response to proteasome inhibitors

The combination of preclinical research and ongoing clinical studies (e.g., add-on studies) will be needed to identify and confirm determinants of resistance and markers for clinical response in order to further personalize the treatment of acute leukemia with PI. Based on accumulating data, prediction of effectivity of PI lies in the composition of the proteasome, in particular the ratio between constitutive- and immunoproteasome subunits. This can either be assessed by measuring protein expression of the different subunits [14, 121] or their specific catalytic activities for which several assays are available [151, 152]. In addition, the activity assays can be used for pharmacodynamic monitoring of PI inhibition and duration of inhibition in PBMC’s [152, 153].

Since mutations in the genes encoding proteasome subunits have not been found in primary MM and acute leukemia patient samples either before or after treatment with PI, they are currently not considered potential biomarkers for resistance to proteasomes. However, when patients are treated for prolonged time periods such as the maintenance treatment of elderly MM patients with IXA, they might be acquired. Therefore, add-on studies measuring the mutational status in samples during treatment are currently performed. If mutations are identified, they may be used to monitor acquired resistance to PI. Selecting the patients that benefit from PI treatment and the recognition of PI resistance is indispensable for the optimal implementation of PI in acute leukemia treatment.
